# Vulnerability pathways to mental health outcomes in children and parents during COVID-19

**DOI:** 10.1007/s12144-021-02459-z

**Published:** 2021-11-19

**Authors:** Jala Rizeq, Daphne J. Korczak, Katherine Tombeau Cost, Evdokia Anagnostou, Alice Charach, Suneeta Monga, Catherine S. Birken, Elizabeth Kelley, Rob Nicolson, Spit for Science, Christie L. Burton, Jennifer Crosbie

**Affiliations:** 1 Department of Psychiatry, The Hospital for Sick Children (SickKids), 555 University Ave, Psychiatry Research, 4th Floor, Black Wing, Toronto, ON M5G 1X8 Canada; 2 Department of Psychiatry, Faculty of Medicine, University of Toronto, Toronto, ON Canada; 3 Department of Pediatrics, Faculty of Medicine, University of Toronto, Toronto, ON Canada; 4 Holland Bloorview Research Institute, Toronto, ON Canada; 5 Division of Paediatric Medicine, Hospital for Sick Children, Toronto, ON Canada; 6 Department of Psychology, Queens University, Kingston, ON Canada; 7 Department of Psychiatry, Western University, London, ON Canada

**Keywords:** Vulnerability, Mental health, Stress, Deprivation, Family functioning

## Abstract

We examined pathways from pre-existing psychosocial and economic vulnerability to mental health difficulties and stress in families during the COVID-19 pandemic. Data from two time points from a multi-cohort study initiated during the COVID-19 pandemic were used. Parents of children 6–18 years completed questionnaires on pre-COVID-19 socioeconomic and demographic factors in addition to material deprivation and stress due to COVID-19 restrictions, mental health, and family functioning. Youth 10 years and older also completed their own measures of mental health and stress. Using structural equation modelling, pathways from pre-existing vulnerability to material deprivation and stress due to COVID-19 restrictions, mental health, and family functioning, including reciprocal pathways, were estimated. Pre-existing psychosocial and economic vulnerability predicted higher material deprivation due to COVID-19 restrictions which in turn was associated with parent and child stress due to restrictions and mental health difficulties. The reciprocal effects between increased child and parent stress and greater mental health difficulties at Time 1 and 2 were significant. Reciprocal effects between parent and child mental health were also significant. Finally, family functioning at Time 2 was negatively impacted by child and parent mental health and stress due to COVID-19 restrictions at Time 1. Psychosocial and economic vulnerability is a risk factor for material deprivation during COVID-19, increasing the risk of mental health difficulties and stress, and their reciprocal effects over time within families. Implications for prevention policy and parent and child mental health services are discussed.

**Supplementary Information:**

The online version contains supplementary material available at 10.1007/s12144-021-02459-z.

The lives of many families have changed drastically in response to the COVID-19 pandemic. As restrictions were imposed to control the spread of the virus, they introduced a cascade of stressors to families (Liu & Doan, 2020), including school closures and material deprivation such as job and service losses, and food insecurity, with some families more vulnerable to this cascade than others. Research shows increased psychological distress and mental health concerns during COVID-19 (e.g., Cost et al., 2021; Feinberg et al., 2021; Kwong et al., 2020; Niedzwiedz et al., 2021; Xiong et al., 2020). Examining the impact of vulnerability factors within the context of COVID-19 is vital in understanding the variability in families’ mental health outcomes. The objective for this study was to empirically examine the direct effect of pre-existing psychosocial and economic vulnerability within families on the material deprivation resulting from COVID-19 restrictions and its indirect impact on parents’ and children’s stress and mental health and family functioning.

Generally, the family system is considered the primary source of support for its members at times of stress (Skinner & Zimmer-Gembeck, 2016). Stress however is not distributed equally across the population; Conditions such as family socioeconomic disadvantage and poverty present as risk factors for cumulative risk exposure – levels of total stress exposure – and health disparities later in life (Evans & Kim, 2012; Pais, 2014). Other factors that increase some families and their children’s vulnerability towards cumulative risk include parenting children with high needs while faced with limited parenting resources, experiencing parental mental health difficulties, family violence, and poor community services or supports (Skinner & Zimmer-Gembeck, 2016). It is especially important to consider vulnerability factors during the COVID-19 pandemic to help us understand children and parents’ response to the restrictions and associated material and financial deprivation posed by the pandemic. Pre-pandemic socioeconomic factors and psychosocial vulnerabilities such as the presence of physical or mental health disability and experiences of marginalization or being part of an ethnic minority group can contribute to some families’ susceptibility to the material deprivation and service disruption caused by COVID-19 (Crawley et al., 2020; Golberstein et al., 2020; Holmes et al., 2020; Jenkins et al., 2020; Prime et al., 2020). Families with pre-existing psychosocial vulnerabilities are likely strained (Hinojosa et al., 2015) and the introduction of further disruption can lead to disproportionate negative consequences, especially when mental health services are halted (Golberstein et al., 2020; Masonbrink & Hurley, 2020).

These vulnerability factors may directly impact the level of financial and material deprivation experienced due to COVID-19 and indirectly impact the associated level of stress and mental health difficulties within the family. High stress and mental health difficulties within the family can also strain families’ capacity to maintain cohesion, communication, effective problem-solving, and emotional involvement, particularly when they are faced with added demands due to material and financial hardship. For example, financial stress and pressure within the family is shown to impact youths’ behaviour and mental health indirectly through parents’ mental health and distress, parenting practices and conflict (e.g., Landers-Potts et al., 2015; Neppl et al., 2016; Ponnet, 2014). Further, parental cultural stress and mental health symptoms negatively impacted family functioning, including low positive parenting, parental involvement, and family cohesion, which in turn were associated with higher substance use and lower self-esteem in youth (Lorenzo-Blanco et al., 2017).

Empirical work during the pandemic has supported some of these pathways. Within the UK, individuals with lower socioeconomic status experienced higher material and financial deprivation during COVID-19 and this inequality in experiences was maintained over time (Wright et al., 2020). Similarly, lower socioeconomic status and pre-existing mental and physical conditions prior to COVID-19 were associated with increased risk of mental health concerns during COVID-19 in adults in the UK (Kwong et al., 2020). Further, maternal mental health symptoms in a Canadian sample increased during COVID-19 in part due to income and childcare service disruption (Racine et al., 2021). Parental mental health and family financial difficulties were also among the risk factors for heightened psychological difficulties in young children during COVID-19 (Moulin et al., 2021). In addition, research has shown that quality of family relationships, including coparenting and parenting quality, deteriorated during the pandemic (Feinberg et al., 2021) and parenting stress and mental health symptoms limited parents’ ability to effectively buffer COVID-19-related stress on children’s mental health (Cohodes et al., 2021).

To our knowledge there are no studies reporting on the reciprocal effect of parent and child mental health and stress due to COVID-19 over time and their impact on general family functioning (i.e., cohesion, problem-solving, communication, and emotional responsiveness in the family). Further, the limited work on vulnerability factors and children’s mental health outcomes within the context of COVID-19 is based on parent report. In this comprehensive study, we examined the effect of pre-existing psychosocial and economic vulnerability as measured by pre-existing child mental health diagnosis, parental education, household income, and child ethnicity on material deprivation due to COVID-19 restrictions. We then examined the impact of material deprivation on children and parents’ stress due to COVID-19 restrictions and mental health difficulties, and the reciprocal effects of these latter variables over two time points. We also assessed the impact of child and parent mental health difficulties and stress on subsequent family functioning. Although the pandemic is a time-limited stressor, which may limit our delineation of developmental processes and change, it offers a unique window to capturing some families’ susceptibility and response to stress and the implications for mental health. Understanding these pathways can offer important information for prevention of mental health difficulties within families and valuable insight to the function of psychosocial and economic factors in children’s and parents’ mental health during times of public health crises.

## Method

### Participants and Procedures

The data used for this study were collected from three cohorts in Ontario with an existing participant base, who collaborated to study the effects of COVID-19 on family mental health. There were two clinically referred mental health and neurodevelopmental cohorts (SickKids Psychiatry and Province of Ontario Neurodevelopmental Disorder (POND) network) and one community cohort (Spit for Science). See supplemental material for description of the separate cohorts.

COVID-19 emergency measures were implemented in Canada in mid-March 2020. Parents who had previously consented to be contacted for research were sent an email about participation in the COVID-19 and Mental Health Study in Ontario. The email also included a separate link to send to their child if their child was between the ages of 10 and 18 years and interested in participating. Parents and children completed the study online using separate links via REDcap in both Time 1 and Time 2. All participants were sent a link to complete Time 1 survey in April 2020 and were sent another link 1 month after completing Time 1 to complete the survey for Time 2. Participants completed Time 1 between May and September 2020 and Time 2 between June and November 2020 with an average interval of 42.2 days (SD = 27.68) between time points.

Data used in this study were from participants with over 50% of all relevant parent-rated variables across two time points (N = 1069 at Time 1 and N = 742 at Time 2). This included data from 441 youth at Time 1 and 215 at Time 2. No bias of attrition was observed (see Table 1 in supplementary material). Participant characteristics as reported by parents at Time 1 are presented in Table 1. There were 1763 registered cases initially. When sibling pairs were present, we removed data associated with the younger sibling (n = 213) to include greatest range of ages, leaving 1550 participants. We also excluded data associated with children 5 years or younger (n = 54) because most of the measures utilized in this study are not validated for that age group, leaving 1496 participants, after which we removed participants with 50% or more missing data on relevant parent-rated variables (n = 427).

**Table 1:** Participant characteristics at Time 1 and 2

	Time 1			Time 2		
	*% (n)*	*Mean (SD)*	*N*	*% (n)*	*Mean (SD)*	*N*
Full Sample, Parent-Report						
Child age (in years)	–	11.06 (3.37)	1069	–	11.15 (3.34)	742
Ethnicity/ancestry of the child			1069			742
European, non-aboriginal North American	59.96% (641)	–		61.05% (453)	–	
Non-European	14.41% (154)	–		12.67% (94)	–	
Multiple ancestries	23.76% (254)	–		24.93% (185)	–	
Did not disclose	1.87% (20)	–		1.35% (10)	–	
Assigned sex of the child			1069			742
Male	57.62% (616)	–		55.66% (413)	–	
Female	42.00% (449)	–		43.94% (326)	–	
Prefer not to respond	0.37% (4)			0.40% (3)	–	
Child/adolescent gender identity			1069			742
Boy	57.34% (613)	–		55.39% (411)	–	
Girl	41.07% (439)	–		42.99% (319)	–	
Trans or non-binary or self-described	1.03% (11)	–		1.21% (9)	–	
Prefer not to answer or identity not listed	0.56% (6)	–		0.40% (3)	–	
Any Premorbid MH/psychiatric diagnosis	65.58% (701)	–	1069	65.50% (486)	–	742
Last year’s family income > $99,999	51.92% (553)	–	1065	52.44% (387)	–	738
Parent 1 education 4-year university degree or higher	74.93% (798)	–	1065	76.02% (561)	–	738
Parent 2 education 4-year university degree or higher	67.38% (566)	–	840	66.39% (393)	–	592

### Measures

#### Parent-Reported Variables

##### Demographics

Parent-reported child age, ethnicity, sex assigned at birth, and gender identity was assessed at Time 1 using questions adapted from the CRISIS questionnaire (Merikangas et al., 2020). The breakdown of these variables is in Table 1.

##### Pre-COVID Psychiatric Child Diagnosis

Parent reported the presence or absence of mental health condition, specifically depression, anxiety, OCD, ADHD, ASD, Intellectual Disability, and other neurodevelopmental conditions. If the parent indicated the presence of any of these mental health conditions, then the variable was coded as 1 (present), and if not as 0 (absent).

##### Socioeconomic Status Prior to COVID-19

The socioeconomic index comprised of 3 items asking about household income in the past year rated on a 6-point scale (1 = < 29,999; 6 = > 200,000) and parental education rated on a 7-point scale for each parent (some grade school; 7 = a graduate or professional degree). Parents’ ratings on each of the items was reversed before summing them to form a total score of socioeconomic status, whereby higher scores indicated higher socioeconomic vulnerability (omega^1^ = .70).

##### Material/Economic Deprivation Due to COVID-19

Endorsement on five items from the CRISIS questionnaire (Merikangas et al., 2020) were summed to present an index of material and economic deprivation due to COVID-19 restrictions. Items asked about food, housing, job, and income stability and disruption as a result of COVID-19 emergency measures (see supplementary for item frequency). Higher total scores were indicative of higher deprivation (omega = .82).

##### Parent Stress Due to COVID-19 Restrictions

Four items from the CRISIS Questionnaire (Merikangas et al., 2020) were used to form an index of parent stress due to COVID-19 restrictions and isolation. Item responses were provided on a five-point Likert scale (1 = not at all; 5 = extremely). Higher total scores indicate a higher level of stress (omega Time 1 = .65 and Time 2 = .72).

##### Parent Depression

The 8-item Patient Health Questionnaire (PHQ-8; Kroenke et al., 2009) was used to assess parent depression. Items were rated on a 4-point likert scale (1 = not at all; 4 = nearly every day). Higher total scores indicate higher depression (omega Time 1 = .90 and Time 2 = .91).

##### Parent Anxiety

The 7-item General Anxiety Disorder (GAD-7; Kroenke et al., 2010) was used to assess parent anxiety. Items were rated on a 4-point likert scale (1 = not at all; 4 = nearly every day). Higher total scores indicate higher anxiety (omega Time 1 = .91 and Time 2 = .92).

##### Child Depression

To rate children’s mood, the ten depression items were used from the Revised Children’s Anxiety and Depression Scale (RCADS; Chorpita et al., 2005). Parents rated the items on a 4-point likert scale (0 = never; 3 = always). Higher scores indicate higher depression (omega Time 1 = .86 and Time 2 = .90).

##### Child Anxiety

Nine items were used from the generalized anxiety subscale of the Screen for Child Anxiety-Related Disorders (SCARED; Monga et al., 2000) to rate children’s anxiety. Items were rated on a 3-point likert scale (0 = not true or hardly true; 2 = very true or often true), Higher total scores indicate higher anxiety (omega Time 1 = .92 and Time 2 = .93).

##### Child Attention and Hyperactivity

Single-item measures from the CRISIS Questionnaire (Merikangas et al., 2020) were used at Time 1 and Time 2 to assess child’s inattention and hyperactivity. Parents were asked to rate child’s ability to concentrate on a 5-point scale (1 = very focused/attentive; 5 very unfocused/distracted). Parents also rated their child’s ability to control restlessness and fidgeting on a 5-point scale (1 = not fidgety/restless at all; 5 = extremely fidgety/restless).

##### Family Functioning

The 12-item general family functioning subscale was used from the McMaster Family Assessment Device (Byles et al., 1988) to assess family functioning at Time 2. Items were rated on a 4-point likert scale (1 = strongly agree; 4 = strongly disagree). A higher total score indicates better general family functioning (omega = .90).

#### Youth-Reported Measures

##### Youth Anxiety

Nine items from the Screen for Child Anxiety-Related Disorders (SCARED; Monga et al., 2000) were used to assess self-reported anxiety in youth. Items were rated on a 3-point likert scale (0 = not true or hardly true; 2 = very true or often true). Higher total scores indicate higher anxiety (omega Time 1 = .89 and Time 2 = .93).

##### Youth Depression

The 20-item Centre for Epidemiologic Studies-Depression scale for Children (CES-DC; Radloff, 1977) was used as a self-report measure for youth depression. Items were rated on a 4-point likert scale (0 = not at all; 3 = a lot). Higher total scores indicate lower mood or higher depression (omega Time 1 = .84 and Time 2 = .94).

##### Youth Stress Due to COVID-19 Restrictions

The index from the CRISIS Questionnaire (Merikangas et al., 2020) used to assess parent stress due to COVID-19 restrictions and isolation was used to assess youth stress due to COVID-19 restrictions (described above). Higher scores indicate higher level of stress (omega Time 1 = .70 and Time 2 = .74).

### Data Analysis

Data were analysed in R Studio version 1.3.1093 (RStudio Team, 2020). For mental health measures, total scale scores were standardised (z-scores) and combined to compute total mental health composite scores. For the purposes of the SEM, child ethnicity was dichotomized with 1 = non-European/North American and multiple and 0 = European, non-Indigenous North American.

First, we calculated descriptive statistics for all variables used in this study. Then confirmatory factor analysis (CFA) was used to test the measurement model of socioeconomic vulnerability and material deprivation variables in our sample, before expanding it to a structural equation model (SEM). Correlations and two SEMs were used to assess the associations among the parent-rated variables across the two time points and parent-rated and youth-rated variables at the two time points. Reciprocal associations between child and parent mental health difficulties and stress due to COVID-19 restrictions across Time 1 and 2 were estimated in the SEM. Further, in the SEM, we tested the indirect paths from pre-existing vulnerability variables (i.e., household socioeconomic status, child ethnicity, and child mental health condition) to child and parent mental health and parent stress due to COVID-19 restrictions at Time 1 through material deprivation due to COVID-19 restrictions.

CFA and SEM models were estimated using the lavaan package (version 0.5–17; Rosseel, 2012). Weighted least squares-mean adjusted estimator with robust standard errors and fit statistics was used. Missing data were handled using a pairwise approach. Model fit was evaluated using the standardized root mean square residual (SRMR), the root mean square error of approximation (RMSEA), the comparative fit index (CFI), and the Tucker-Lewis index (TLI).

## Results

### Descriptive Statistics and Correlations

Table 2 presents the descriptive statistics for total scale and composite scores. Bivariate correlations among the variables in Time 1 and 2 can be found in the supplementary material (Tables 3 and 4). At Time 1, pre-existing socioeconomic vulnerability was significantly positively associated with material and economic deprivation and parent stress due to COVID-19 restrictions, parent mental health scores and parent-rated child mood, inattention, and fidgety behaviours with correlations ranging between *r* = .08 and .26 (all *ps* < .05), but not with parent-rated child anxiety *(r* = .03, *p* = .38). Material and economic deprivation due to COVID-19 was significantly positively associated with parent stress due to COVID-19, parent mental health scores, and parent-rated child mental health scores (*rs* between .12 and .41, *ps* < .05). Parent and youth-rated variables including parent and child stress due to COVID-19 and mental health concerns were all significantly and positively correlated (*ps* < .05). At Time 2, all parent-and youth-rated variables, including parent and child stress due to COVID-19 and mental health concerns were all significantly positively correlated (*ps* < .05) and negatively correlated with family functioning (*rs* between −.17 and − .34, *ps* < .05).

**Table 2:** Descriptive statistics for parent and child/youth-reported total scores across Time 1 and 2

Variable	n	Mean	SD	Range (min, max)
Time 1 – parent reported				
Parent stress due to COVID-19 restrictions	1065	9.93	2.96	4,20
Parent mood	992	15.60	5.47	8,32
Parent anxiety	992	13.74	5.07	7,28
Parent total mental health*	992	0	1.87	−2.72,5.81
Child mood	1062	7.50	5.49	0,30
Child anxiety	1059	7.27	5.15	0,18
Child attention	1027	3.42	1.17	1,5
Child fidgety	1027	2.75	1.19	1,5
Child total mental health*	1015	−0.01	3.04	−6.32,8.33
Time 2 – parent reported				
Parent stress due to COVID-19 restrictions	585	9.27	3.05	4,20
Parent mood	555	14.48	5.37	8,32
Parent anxiety	555	12.78	5.04	7,28
Parent total mental health*	555	0	1.88	−2.35,6.28
Family functioning	655	37.93	5.71	19,48
Child mood	577	7.49	5.54	0,26
Child anxiety	572	7.47	5.35	0,18
Child attention	575	3.18	1.11	1,5
Child fidgety	575	2.63	1.17	1,5
Child total mental health*	561	−0.03	3.04	−6.10,7.70
Time 1 – youth reported				
Youth stress due to COVID-19 restrictions	441	9.73	3.39	4,20
Youth mood	433	21.29	12.83	0,57
Youth anxiety	434	8.39	5.24	0,18
Youth total internalizing*	433	0	1.83	−3.26,4.39
Time 2 – youth reported				
Youth stress due to COVID-19 restrictions	215	8.76	3.36	4,18
Youth mood	207	20.22	13.16	0,52
Youth anxiety	210	8.01	5.70	0,18
Youth total internalizing*	207	−0.01	1.89	−2.94,4.17

*Note.* * indicates composite z-score

### CFA and SEM Results

The two-factor model with socioeconomic status and material and economic deptivation due to COVID-19 factors fit the data well (CFI = .97, TLI = .96, RMSEA = .07, SRMR = .08). The completely standardized factor loadings for both factors were significant (all *ps* < .001) and ranged from .52 to .84 on the socioeconomic index factor and .59 to .93 on the material and economic deprivation due to COVID-19 restrictions factor. The two factors were postively and significantly correlated *(r* = .44, *p* < .001).

**Fig. 1:**
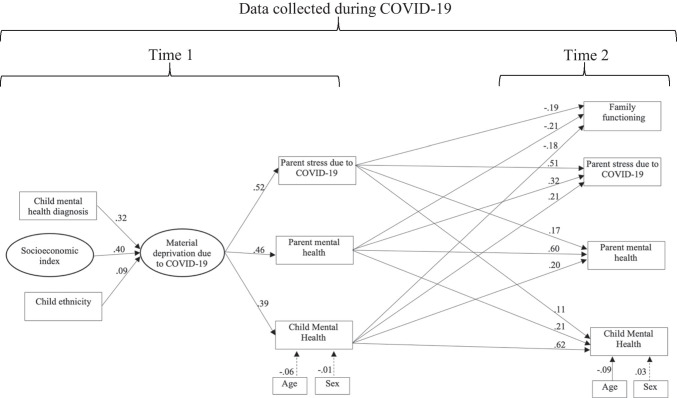
Structural equation model with parent-rated variables. *Note.* Dashed lines indicate non-significant regression coefficients (*p* > .05). To reduce clutter, the figure omits the observed item-level indicators of the material deprivation and socioeconomic vulnerability index factors and the residual correlations among the endogenous variables at Time 2

The SEM shown in Fig. 1 using parent-reported variables fit the data adequately (CFI = .90, TLI = .92, RMSEA = .07, SRMR = .08). The completely standardized regression coefficients are in Fig. 1. Socioeconomic vulnerability factor, presence of pre-existing child mental health diagnosis, and child ethnicity “non-European or multiple” signficantly predicted higher material deprivation due to COVID-19 at Time 1 (all *ps* < .05). Material deprivation at Time 1 was positively and significantly associated with parent mental health difficulties, parent stress due to COVID-19 restrictions, and child mental health difficulties (while controlling for child age and sex) at Time 1 (all *ps* < .05). As shown in Table 3, the indirect effects from pre-existing vulnerability factors to parent and child mental health difficulties and parent stress due to COVID-19 restrictions at Time 1 through material deprivation due to COVID-19 were all significant. Higher parent and child mental health difficulties and parent stress due to COVID-19 restictions at Time 1 significantly predicted worse family functioning at Time 2 (all *ps* < .05). Parent and child mental health difficulties and parent stress due to COVID-19 restrictions demonstrated both significant stability and reciprical effects between Time 1 and 2 (all *ps* < .05).

**Table 3:** Indirect effects in the SEM with parent-rated variables

Outcome	Indirect path	Standardized regression coefficient	*p*
Parent stress due to COVID-19	Socioeconomic index * material deprivation due to COVID-19	.21	<.001
	Child mental health diagnosis * material deprivation due to COVID-19	.16	<.001
	Child ethnicity * material deprivation due to COVID-19	.05	.011
Parent mental health	Socioeconomic index * material deprivation due to COVID-19	.18	<.001
	Child mental health diagnosis * material deprivation due to COVID-19	.15	<.001
	Child ethnicity * material deprivation due to COVID-19	.04	.010
Child mental health	Socioeconomic index * material deprivation due to COVID-19	.15	<.001
	Child mental health diagnosis * material deprivation due to COVID-19	.12	<.001
	Child ethnicity * material deprivation due to COVID-19	.04	.011

At Time 1, the model accounted for 25.7% of the variance in material and economic deprivation due to COVID-19 restrictions, 27.1% in parent stress due to COVID-19 restrictions, 21.3% in parent mental health, and 15.1% in child mental health. At Time 2, the model accounted for 15.5% of the variance in family functioning and 55.2%, 53.2%, and 53.0% of the variance in parent stress due to COVID-19 restrictions, parent mental health, and child mental health respectively.

**Fig. 2:**
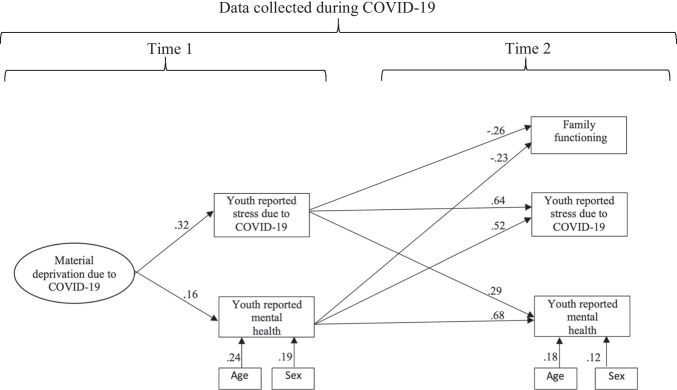
Truncated structural equation model with parent-rated and youth-rated variables. *Note.* All regression coefficients were significant (*p* < .05). To reduce clutter, the figure omits the observed item-level indicators of the material deprivation factor and the residual correlations among the endogenous variables at Time 2

We also estimated a truncated SEM with youth-reported mental health and stress due to COVID-19 restrictions at Time 1 and 2 and parent-rated material deprivation due to COVID-19 restrictions at Time 1 and parent-rated family functioning at Time 2.^2^ The model fit the data adequately (CFI = .95, TLI = .95, RMSEA = .06, SRMR = .10). The completely standardized regression coefficients are in Fig. 2. Parent-reported material deprivation at Time 1 was significantly positively associated with youth reported mental health difficulties (while controlling for child age and sex) and stress due to COVID-19 restictions at Time 1 (*ps* < .05). Higher youth reported mental health difficulties and stress due to COVID-19 restictions scores at Time 1 significantly predicted worse parent-rated family functioning at Time 2. They also showed significant stability and reciprocal effects across Time 1 and 2 (*ps* < .05).

At Time 1, the model accounted for 10.1% and 12.5% of the variance in youth-reported stress due to COVID-19 restrictions and mental health difficulties, respectively. At Time 2, the model accounted for 12.7% of the variance in parent-reported family functioning and 71.3% and 70.5%, of the variance in youth-reported stress due to COVID-19 restrictions and mental health difficulties, respectively.

## Discussion

In this study we identified pathways from pre-existing psychosocial and economic vulnerability to mental health difficulties in children and parents during COVID-19. Specifically, higher psychosocial and economic vulnerability predicted higher material deprivation due to COVID-19 restrictions, which in turn was significantly associated with the sequelae of child and parent mental health difficulties and stress due to COVID-19 restrictions. The reciprocal associations were significant between child and parent mental health and stress due to COVID-19 restrictions across the two time points. Family functioning at follow up was also significantly negatively impacted by child and parent mental health difficulties and stress at the first time point. These results have important implications for clinical work and policy to support family functioning and the wellbeing of children and parents.

The SEM demonstrates that families with pre-existing psychosocial and economic vulnerability are more likely to be disadvantaged in the face of COVID-19. Specifically, the presence of a diagnosed mental health condition in the child and being of a non-European ethnic origin or of multiple ethnic origins and a family with lower socioeconomic status uniquely predicted higher material deprivation due to COVID-19, extending work by Wright and colleagues with adults in the UK (2020). The findings are also consistent with work demonstrating a negative association between socioeconomic status and COVID-19 related economic trauma and routine disturbance (Kira, Shuwiekh, Alhuwailah, et al., 2021a). These associations highlight the strain various pre-existing vulnerabilities such as having a child with disability place on families, which is exacerbated in times of public health crisis (Prime et al., 2020). Child ethnicity had a weak effect on material deprivation, suggesting that it is not a strong determinant of material deprivation in our sample. Further, higher material deprivation due to COVID-19 restrictions was associated with higher parent stress due to restrictions and mental health concerns, consistent with associations reported outside of the context of COVID-19 (e.g., Neppl et al., 2016; Ponnet, 2014). Importantly, material deprivation as reported by parents was also associated with higher youth reported stress due to restrictions and mental health concerns. This finding follows from work showing that weakening of state economy is associated with worsening mental health in children and youth (Golberstein et al., 2019), suggesting that proximal hardship and associated deprivation may be important when examining short-term mental health. The indirect effects of the three pre-existing vulnerability factors on parent and child mental health difficulties and parent stress due to COVID-19 restrictions through material deprivation due to COVID-19 were also significant. These pathways demonstrate the cascading effect of early vulnerabilities and the associated mental health and stress outcomes within families. Our findings support recommendations that at times of crisis and weak economic conditions, policy responses should include financial and material supports, to reduce the financial burden, and eventually benefit child and parents’ mental health (Golberstein et al., 2019). Abundant evidence exists to support targeting socioeconomic determinants of health, especially with children and families to mitigate cascading adversity that increase the likelihood that children will experience poor outcomes in childhood and adulthood (see Thornton et al., 2016).

The significant reciprocal associations demonstrate that both parent and child mental health impact each other at later time points, highlighting their reciprocal contribution over time and reinforcing the importance of parent engagement in child services (Brody et al., 2013; Haine-Schlagel & Walsh, 2015). Additionally, identifying and targeting protective factors and enhancing coping for parents impacted by material deprivation is vital (Landers-Potts et al., 2015). Notably, the reciprocal effects between stress due to COVID-19 restrictions and child and parent mental health difficulties are also significant, with stronger paths from mental health difficulties to stress than from stress to mental health. These findings underscore the increased likelihood of experiencing heightened stress due to restrictions over time as a function of mental health difficulties; Therefore, addressing mental health symptoms may improve parents’ and children’s ability to buffer the stress of the pandemic. Together, in addition to mental health service provision, families would likely benefit from strategies to help reduce stress and offer opportunities to socialize and engage with others responsibly and in accordance with public health guidelines.

Both higher parent and youth-rated mental health difficulties and stress due to COVID-19 restrictions predicted worse general family functioning as rated by parents at Time 2, in line with expectations that increased distress and mental health difficulties within the family could negatively impact family functioning. With further waves of data collection, we will be able to delineate the reciprocal effect of family functioning and parent and child mental health and stress due to COVID-19 restrictions. Nonetheless, it appears that increased levels of stress and mental health negatively impact the degree of cohesion, problem-solving, communication, and emotional responsiveness in the family. Researchers warn against potential long term psychological scarring within families and the persistence of their negative sequelae beyond the pandemic (Feinberg et al., 2021). They further highlight the urgent need for family-based interventions especially for families with pre-existing vulnerabilities (Fontanesi et al., 2021). Specifically, the authors identified the need to improve supportive resources to families, accessibility to child protection and mental health services, informed child and family policy, and the development of best practice guidelines and training opportunities for professionals and service providers.

The strengths of this study lie in its inclusion of both parent and youth report, longitudinal design, and sampling of participants with pre-existing mental health conditions. Despite the important contributions of this study, several limitations should be noted. We focused on the negative sequelae of material deprivation due to COVID-19 restrictions to the exclusion of protective factors that may have moderated some of the pathways explored. Certainly, considering the interplay between protective and risk factors will be important to accurately capture children and parents’ response to stressful life events (Cicchetti, 2010). Service provision and utilization during the COVID-19 pandemic is an important area for future study in order to elucidate some factors that may mitigate the negative consequences of COVID-19-related stress and deprivation on families’ mental health. Further, our sample is limited in its under-representation of low-income families. It is possible that a more variable socioeconomic composition would have shown a stronger association between pre-existing vulnerability and material deprivation, and in turn differentially impacting the pathways to stress and mental health. In addition, we did not account for the effect of neighbourhood deprivation, which will be an important factor to consider in future work. Another relevant outcome to assess in future work is traumatic stress, as various COVID-19 related traumas have been identified (Kira, Shuwiekh, Rice, et al., 2021b) and traumatic stress symptoms have been associated with COVID-19 related worries and social isolation (Boyraz et al., 2020). Finally, our sample attrition rate was high between Time 1 and 2 but the results did not suggest a bias of attrition.

### Conclusion

Taken together, the findings from this study have both clinical and policy implications, demonstrating that a global health crisis’ effect extends to households, indirectly impacting family functioning, and stress and mental health of both children and parents. Preventative interventions for children’s and parent’s mental health are essential at times of public crises, particularly for groups with pre-existing mental health conditions and low socioeconomic status, to mitigate perceived stress and reduce the strain on family functioning. Importantly, Policy responses to public crises should include material and financial support to buffer the impact of crisis-related material deprivation on mental health and stress within the family. Continued research in this area to delineate temporal pathways from early vulnerability to cumulative stress and mental health difficulties is important for the allocation and provision of early intervention and prevention. Future research would benefit from assessing the effect of protective factors and access to services on those pathways.

## Supplementary Information

ESM 1 (DOCX 29 kb)

## Footnotes

1 Reliability was estimated using McDonald’s coefficient omega (McDonald, 1999) for all of the total scores used in this research project because it does not assume “essential tau equivalence,” which implies equal factor loadings across items.

2 We did not include the pre-existing factors in the model because the associations would be redundant with what is presented in the first SEM reported in Figure 1.
