# Supplementary material for: Vulnerability pathways to mental health outcomes in children and parents during COVID-19
Source: Curr Psychol. 2021 Nov 19:1–11. Online ahead of print. doi: 10.1007/s12144-021-02459-z (PMC8603653; doi:10.1007/s12144-021-02459-z)
Supplement: Supplementary file 1 — (DOCX 29 kb) [file 12144_2021_2459_MOESM1_ESM.docx]

**Supplemental Materials**

**Description of the three research cohorts.**

**Supplemental Table 1.** Comparisons on major outcomes at Time 1 across participants who completed Time 2 and those who did not.

**Supplemental Table 2.** Response frequencies for material and economic deprivation due to COVID-19 restrictions items reported at Time 1

**Supplemental Table 3.** Correlations among variables measured in Time 1

**Supplemental Table 4.** Correlations among variables measured in Time 2

**Description of the three research cohorts.**

**SickKids Psychiatry**

SickKids is the largest children’s hospital in Canada with over 1,800 new referrals to the Department of Psychiatry annually. Children and youth (ages 4 to 17 years) referred for mental health assessment are from diverse socioeconomic backgrounds from within and outside of the Greater Toronto Area (GTA). Following referral through the centralized intake process, all children and families complete standardized measures of mental health symptoms and functional impairment and are offered the opportunity to participate in the ongoing and future research activities within the Department, including participation in a clinical research registry.

**POND network**

Research network focused on studying the biology and underpinning of neurodevelopmental disorders. It primarily recruits Children/adolescents 6-18 years with neurodevelopmental disorders, including autism spectrum disorders (ASD), ADHD, OCD, and intellectual disability.

<https://pond-network.ca/>

**Spit for Science**

A population-based sample of children/adolescents 6-18 years recruited at an urban science museum.

<https://lab.research.sickkids.ca/schachar/spit-for-science/>

Supplemental Table 1. Comparisons on major outcomes at Time 1 across participants who completed Time 2 and those who did not.

|  | **Group that completed Time 2** | **Group that did not complete Time 2** |  |  |
| --- | --- | --- | --- | --- |
| **Variable at Time 1** | **Mean (SD)** | **Mean (SD)** | **t (df)** | **P value** |
| Parent reported stress due to COVID-19 | 9.83 (2.96) | 10.15 (2.98) | 1.62 (620.68) | .1058 |
| Parent reported parent mental health composite | 0.03 (1.87) | -0.09 (1.88) | -0.86 (439.83) | 0.3901 |
| Parent reported child mental health composite | 0.04 (3.12) | -0.13 (2.83) | -0.83 (555.40) | .4078 |
| Youth reported stress due to COVID-19 | 9.81 (3.46) | 9.35 (3.05) | -1.19 (133.17) | .2351 |
| Youth reported child mental health composite | 0.00 (1.88) | 0.00 (1.54) | 0.03 (129.94) | .9741 |

Note. Welch t-test was used because of non-homogenous variance across groups.

Supplemental Table 2. Response frequencies for material and economic deprivation due to COVID-19 restrictions items reported at Time 1

| **Variable** | **n** | **Qualifier (% of sample)** |
| --- | --- | --- |
| Food insecurity | 1065 | Yes (9.86%) |
| Living situation stability | 1065 | Moderately to extremely worried (27.70%) |
| Financial instability | 1065 | Moderately to extremely affected (35.64%) |
| Job loss | 1069 | Yes (12.16%) |
| Reduced ability to earn money | 1069 | Yes (23.95%) |

Supplemental Table 3. Correlations among variables measured in Time 1

| Variable | 1 | 2 | 3 | 4 | 5 | 6 | 7 | 8 | 9 | 10 | 11 | 12 | 13 | 14 | 15 |
| --- | --- | --- | --- | --- | --- | --- | --- | --- | --- | --- | --- | --- | --- | --- | --- |
| Parent reported |  |  |  |  |  |  |  |  |  |  |  |  |  |  |  |
| 1.Socioeconomic index | 1 |  |  |  |  |  |  |  |  |  |  |  |  |  |  |
| 2. Material deprivation | 0.26 | 1 |  |  |  |  |  |  |  |  |  |  |  |  |  |
| 3.Parent stress due to COVID-19   restrictions | 0.11 | 0.41 | 1 |  |  |  |  |  |  |  |  |  |  |  |  |
| 4.Parent mood | 0.16 | 0.32 | 0.47 | 1 |  |  |  |  |  |  |  |  |  |  |  |
| 5.Parent anxiety | 0.08 | 0.29 | 0.46 | 0.75 | 1 |  |  |  |  |  |  |  |  |  |  |
| 6.Parent mental health* | 0.13 | 0.32 | 0.5 | 0.93 | 0.93 | 1 |  |  |  |  |  |  |  |  |  |
| 7.Child mood | 0.11 | 0.22 | 0.37 | 0.39 | 0.35 | 0.4 | 1 |  |  |  |  |  |  |  |  |
| 8.Child anxiety | 0.03^ | 0.18 | 0.28 | 0.28 | 0.33 | 0.33 | 0.57 | 1 |  |  |  |  |  |  |  |
| 9.Child attention | 0.11 | 0.12 | 0.27 | 0.26 | 0.28 | 0.29 | 0.49 | 0.27 | 1 |  |  |  |  |  |  |
| 10.Child Fidgety | 0.09 | 0.18 | 0.34 | 0.35 | 0.36 | 0.38 | 0.44 | 0.3 | 0.55 | 1 |  |  |  |  |  |
| 11.Total child mental health* | 0.11 | 0.23 | 0.42 | 0.42 | 0.43 | 0.45 | 0.82 | 0.7 | 0.76 | 0.75 | 1 |  |  |  |  |
| Youth reported |  |  |  |  |  |  |  |  |  |  |  |  |  |  |  |
| 12. Youth stress due to COVID-19   restrictions | 0.05^ | 0.23 | 0.39 | 0.27 | 0.31 | 0.3 | 0.32 | 0.3 | 0.18 | 0.24 | 0.34 | 1 |  |  |  |
| 13. Youth anxiety | -0.04 | 0.1 | 0.16 | 0.18 | 0.21 | 0.21 | 0.4 | 0.57 | 0.12 | 0.13 | 0.41 | 0.37 | 1 |  |  |
| 14.Youth depression | 0.0^ | 0.11 | 0.24 | 0.24 | 0.22 | 0.24 | 0.57 | 0.48 | 0.22 | 0.15 | 0.47 | 0.45 | 0.67 | 1 |  |
| 15.Youth total internalizing* | -0.02 | 0.12 | 0.22 | 0.23 | 0.23 | 0.24 | 0.53 | 0.58 | 0.19 | 0.15 | 0.48 | 0.45 | 0.91 | 0.91 | 1 |

*Note.* * indicates composite z-score. ^ indicates a non-significant correlation with *p* > .05.

Supplemental Table 4. Correlations among variables measured in Time 2

|  | 1 | 2 | 3 | 4 | 5 | 6 | 7 | 8 | 9 | 10 | 11 | 12 | 13 | 14 |
| --- | --- | --- | --- | --- | --- | --- | --- | --- | --- | --- | --- | --- | --- | --- |
| Parent reported |  |  |  |  |  |  |  |  |  |  |  |  |  |  |
| 1.Parent stress due to COVID-19   restrictions | 1 |  |  |  |  |  |  |  |  |  |  |  |  |  |
| 2.Parent mood | 0.46 | 1 |  |  |  |  |  |  |  |  |  |  |  |  |
| 3.Parent anxiety | 0.47 | 0.76 | 1 |  |  |  |  |  |  |  |  |  |  |  |
| 4.Parent mental health* | 0.5 | 0.94 | 0.94 | 1 |  |  |  |  |  |  |  |  |  |  |
| 5.Child mood | 0.42 | 0.45 | 0.38 | 0.44 | 1 |  |  |  |  |  |  |  |  |  |
| 6.Child anxiety | 0.34 | 0.35 | 0.34 | 0.36 | 0.6 | 1 |  |  |  |  |  |  |  |  |
| 7.Child attention | 0.25 | 0.33 | 0.3 | 0.34 | 0.45 | 0.26 | 1 |  |  |  |  |  |  |  |
| 8.Child Fidgety | 0.34 | 0.36 | 0.36 | 0.38 | 0.48 | 0.32 | 0.52 | 1 |  |  |  |  |  |  |
| 9.Total child mental health* | 0.44 | 0.49 | 0.45 | 0.5 | 0.83 | 0.72 | 0.73 | 0.76 | 1 |  |  |  |  |  |
| 10. Family functioning | -0.29 | -0.33 | -0.3 | -0.34 | -0.25 | -0.25 | -0.25 | -0.17 | -0.31 | 1 |  |  |  |  |
| Youth reported |  |  |  |  |  |  |  |  |  |  |  |  |  |  |
| 11. Youth stress due to COVID-  19 restrictions | 0.42 | 0.24 | 0.3 | 0.28 | 0.37 | 0.29 | 0.31 | 0.34 | 0.41 | -0.2 | 1 |  |  |  |
| 12. Youth anxiety | 0.19 | 0.28 | 0.26 | 0.28 | 0.6 | 0.62 | 0.26 | 0.26 | 0.54 | -0.22 | 0.42 | 1 |  |  |
| 13.Youth depression | 0.18 | 0.22 | 0.22 | 0.23 | 0.7 | 0.56 | 0.29 | 0.26 | 0.57 | -0.19 | 0.5 | 0.78 | 1 |  |
| 14.Youth total internalizing* | 0.19 | 0.26 | 0.25 | 0.27 | 0.69 | 0.63 | 0.29 | 0.27 | 0.59 | -0.22 | 0.49 | 0.94 | 0.94 | 1 |

*Note.* * indicates composite z-score. All correlations are significant at *p* < .05.
